# Herbal Medicine as an Alternative Medicine for Treating Diabetes: The Global Burden

**DOI:** 10.1155/2014/596071

**Published:** 2014-08-18

**Authors:** Geeta Watal, Preeti Dhar, Sharad Kr. Srivastava, Bechan Sharma

**Affiliations:** ^1^Department of Chemistry, University of Allahabad, Allahabad 211002, India; ^2^Department of Chemistry, 1 Hawk Drive, State University of New York, New Paltz, NY 12561, USA; ^3^National Botanical Research Institute, Lucknow 226001, India; ^4^Department of Biochemistry, University of Allahabad, Allahabad 211002, India

Diabetes mellitus (DM) affects 150 million people worldwide including 50 million in India. The incidence rate of the disease is expected to double by 2025. Diabetes emerges due to several factors such as inhibition of glucose absorption, increase in glucose uptake and upregulation of glucose transporters, activation of the nuclear receptor PPAR*γ*, increase in adiponectin release, glycogen metabolism, absent or decreased insulin production and/or impaired function, insulin mimetic and insulinotropic effect, elevation of D-Chloroinositol, incretin mimetics and incretin enhancers, and the role of endogenous opioids on glucose homeostasis and antioxidants. Based on the pancreatic effect, DM is classified as type 1 (insulin dependent diabetes mellitus (IDDM)) and type 2 (noninsulin dependent diabetes mellitus (NIDDM)). DM induced hyperglycemia leads to many clinical complications either at the macrovascular level causing coronary artery and cerebrovascular diseases or at the microvascular level causing renal failure, blindness, limb amputation, neurological complications, and premature death [1, 2]. The symptoms of DM include severe hypoglycemia, lactic acidosis, idiosyncratic liver cell injury, permanent neurological deficit, digestive discomfort, headache, and dizziness.

Existing therapeutics against DM mainly include synthetic hypoglycemic drugs and insulin. These anti-DM drugs usually target a single metabolic pathway to regulate hyperglycemia and are laced with numerous side effects and their efficacies are questionable. Therefore, it is imperative to develop new therapeutic paradigms that can act on more than one key metabolic pathway concerning carbohydrate, fat, or protein metabolism. Herbal preparations from over 200 traditional plants and their bioactive constituents are known to possess antidiabetic property as demonstrated by various screening methods. The extracts or constituents of some plants may act at different levels such as inhibiting glucose absorption from intestines, increasing insulin secretion from the pancreas, enhancing glucose uptake by adipose and muscle tissues, or inhibiting glucose production from hepatocytes [2]. Accordingly, there are ample possibilities to exploit phytochemicals as effective alternative medicines with limited or no side effects.

Given that limited information is available regarding the evidence-based therapeutic usage of many antidiabetic plants, this special issue was proposed. It attracted many submissions; following a peer review, only seven submissions were recommended for publication. These articles include review articles and technical papers that provide comprehensive reports on antidiabetic plants and the mode of action of their active ingredients.

The review article “*Botanical, pharmacological, phytochemical, and toxicological aspects of the antidiabetic plant Bidens pilosa* L.” written by W.-C. Yang provides up-to-date information on the pharmacology, phytochemistry, and toxicology of* Bidens pilosa *in regard to type 1 and type 2 diabetes. The author has highlighted medicinal properties of ingredients isolated from the plant,* B. pilosa* L., which is easy-to-grow, widespread, and a palatable perennial. The author has presented the structure and biosynthesis of* B. pilosa* and its polyynes in relation to their antidiabetic action and mechanism. In the article “*Antidiabetic effect of oral borapetol B compound, isolated from the plant Tinospora crispa*,* by stimulating insulin release,*” F. E. Lokman et al. have presented an evaluation of the antidiabetic property of a biologically active small compound borapetol B (C1) isolated from* T. crispa* in normoglycemic control using Wistar (W) and spontaneously type 2 diabetic Goto-Kakizaki (GK) rats. They found that an acute oral administration of the compound significantly improves blood glucose levels in the treated group in comparison to the placebo group. They observed that plasma insulin levels were significantly enhanced by 2-fold in treated W and GK rats compared to the placebo group at 30 minutes. Their study provides evidence that borapetol B (C1)'s antidiabetic property is mainly due to stimulation of insulin release.

N. H. M.-Radzman et al. in the paper “*Stevioside from Stevia rebaudiana Bertoni increases insulin sensitivity in 3T3-L1 adipocytes*” have reported that stevioside from* Stevia rebaudiana *may exert an antihyperglycemic effect on both rat and human subjects. Using methods such as the radioactive glucose uptake assay, they have shown the improvements in insulin sensitivity in 3T3-L1 cells by elevation of glucose uptake as a result of stevioside treatments. They found that stevioside managed to increase uptake activities to a maximum of 2.08 times (*P* < 0.001) under normal conditions and up to 4.40 times (*P* < 0.001) in insulin-resistant states. It was noncytotoxic to the 3T3-L1 cells. The authors have shown that stevioside can have a direct effect on 3T3-L1's insulin sensitivity, by means of the glucose uptake elevation and the increase in expression of proteins in the insulin-signaling pathway. The article “*Potential roles of Stevia rebaudiana* Bertoni* in abrogating insulin resistance and diabetes: A review*” by N. H. M.-Radzman et al. highlights the mechanisms involving free fatty acids, adipocytokines such as TNF*α* and PPAR*γ* and serine kinases like JNK and IKK*β*, asserted to be responsible for the development of insulin resistance. The review article “*Use of laser-induced breakdown spectroscopy for the detection of glycemic elements in Indian medicinal plants*” by P. K. Rai et al. describes how a unique combination of physics, chemistry, and biological techniques can be exploited to evaluate antidiabetic Indian medicinal plants. They show laser-induced breakdown spectroscopy (LIBS) as a sensitive optical technique being widely used for the detection of glycemic elements from medicinal plants.

G. Sartore et al. in the article “*Mediterranean diet and red yeast rice supplementation for the management of hyperlipidemia in statin-intolerant patients with or without type 2 diabetes*” found that the lipid profile can be modified by consuming Mediterranean diet (MD) and red yeast rice (RYR). They indicate that MD counseling alone is effective in reducing low density lipoprotein (LDL) cholesterol levels in moderately hypercholesterolemic statin-intolerant patients with a presumably low cardiovascular risk, and combining MD with the administration of RYR improves patients with LDL cholesterol levels considerably more than when the patient is on MD only.

In a quest to cure many serious diseases using more cost-effective and safer drugs, there has been a paradigm shift in drug discovery that involves using plant-based molecules. More effort, however, is required to explore newer and more promising herbal ingredients that qualify absorption, distribution, metabolism, and excretion (ADME) criteria with a relatively larger therapeutic index.

*Geeta Watal* *Geeta Watal* *Preeti Dhar* *Preeti Dhar* *Sharad Kr. Srivastava* *Sharad Kr. Srivastava* *Bechan Sharma* *Bechan Sharma*
